# Supplementary material for: Recent genetic drift in the co-diversified gut bacterial symbionts of laboratory mice
Source: bioRxiv. 2024 Aug 14:2024.08.14.607958. Preprint. [Version 1] doi: 10.1101/2024.08.14.607958 (PMC11343198; doi:10.1101/2024.08.14.607958)
Supplement: Supplement 11 [file NIHPP2024.08.14.607958v1-supplement-11.pdf]

Supplementary Information

Nanopore metagenomic sequencing of wild-derived Peromyscus lineages

We deeply sequenced the metagenomes of six species/subspecies of *Peromyscus*, including, sampled at the *Peromyscus* stock center at the University of South Carolina, Columbia. Seven MinION flow cells were used to sequence DNAs extracted from fecal samples, with one flow cell dedicated to fecal samples from each individual host. The final *Peromyscus* dataset contained 30,612,212 reads, ranging from 2,325,236 to 6,018,007 reads per sample. The average per-sample read length ranged from 3,494 to 8,335 base pairs. Metadata for all *Peromyscus* samples analyzed in this study are presented in Extended Data Table 1.

Long-read assembly of bacterial genomes from Peromyscus metagenomes

Assembling and binning contigs from long-read metagenomes generated by Nanopore sequencing of fecal samples from the *Peromyscus* species yielded a total 504 metagenome assembled genomes (MAGs) of high-quality (> 50% completeness < 5% contamination) from the six host species. Bacterial diversity represented in these MAGs spanned 10 phyla, including Actinobacteriota, Bacteriodota, Campylobacterota, Deferribacterota, Desulfobacterota, Firmicutes, Patescibacteria, Proteobacteria, Spirochaetota, and Verrucomicrobiota. Taxonomic assignments of all MAGs newly generated by this study are presented in Extended Data Table 2.

Phylogenetic analyses of rodent MAGs and hosts

Single copy bac120 core genes from each MAG were identified and aligned with GTDB-Tk, and the alignment was used for phylogenetic inference with IQTree2 v2.2.0.4 with the following parameters: --seed 0 -B 1000 -alrt 1000 -mset WAG,LG. *P. maniculatus sonoreinsis* was not

available in the timetree.org database and was therefore placed manually in the host phylogeny as sister to *P. maniculatus bardii* with a divergence time of 500,000 years.

Sensitivity analyses of co-diversification results

We performed a sensitivity analysis to assess the impact of the MAGs from each individual host species by conducting the Hommola co-diversification scan on each possible subset of MAGs containing all MAGs except those from an individual host species. This analysis tested whether the results observed in Fig. 1 depended on MAGs from any individual host species. The results show that the detection of most co-diversifying clades was robust to the exclusion of MAGs from any individual host species (Extended Data Figure 4). The exclusion of MAGs from *Mus musculus domesticus* or *Rattus norvegicus*—the two host species represented by the most MAGs—had the largest impact on the number of co-diversifying clades detected. However, even when MAGs from one of these host species were excluded, scans identified > 100 co-diversifying clades.

Calibration of molecular clocks in the rodent gut microbiota corroborates co-diversification

Given the phylogenetic evidence that symbiont lineages and host species co-diversified, the known divergence dates of host species based on molecular data and fossils can be used to calibrate bacterial molecular clocks[59–61], which are otherwise difficult to calibrate due to the lack of a bacterial fossil record. Symbiont and host evolutionary distances within co-diversifying clades were positively associated in all bacterial families (Families containing > 2 co-diversifying clades are shown in Extended Data Figure 5. All data are presented in Extended Data Table 4), enabling calibration of the rates of molecular evolution in diverse GM taxa. Rates ranged from 0.00163 to 0.00596 substitutions per million years. These rates are within the range

estimated previously from codiversifying symbionts in primates[10] and timeseries data of bacterial pathogens[62–64], further supporting the concurrent diversification of bacterial and rodent host lineages.

Identification of clades ancestral to Muridae

We identified all co-diversifying clades that contained representatives from house mice, a non-house mouse murid and at least one outgroup to the Muridae (40 non-nested, *i.e.*, independent, clades). Identifying these clades allowed us to generate a set of clades ancestral to Muridae independent of data from house mice, thereby enabling us to assess rates of extinction and retention of these clades from either laboratory or wild house mice using a common set of ancestral co-diversifying clades.

Differentially abundant gene families between co-diversifying and non-co-diversifying clades

To identify gene families enriched or depleted in co-diversifying rodent symbionts relative to non-codiversifying rodent gut bacteria independent of bacterial phylogenetic history, we annotated each MAG using the Kyoto Encyclopedia of Genes and Genomes (KEGG) ontogeny. Next, we employed phylogenetic ANOVA[51] using the rodent gut bacterial phylogeny to identify annotations over- or under-represented in co-diversifying gut bacteria ($r > 0.75$, p-value < 0.01) relative to non-co-diversifying gut bacteria ($r < 0$). No individual annotation reached significance after correction for multiple testing. These analyses provided a rank order list of gene annotations significantly associated (positively or negatively) with co-diversification (Extended Data Table 7).

Tests for gain and loss of functions in genomes of laboratory–house mouse symbionts

In addition to adaptive evolution of protein sequences, gut bacterial genomes can adapt to novel environments through changes in gene content. Genes that benefit fitness in the new environment can be gained by gene duplication or horizontal transfer and favored in bacterial populations by positive selection, whereas ancestral genes that no longer provide appreciable fitness benefits can be deleted by mutation (which displays a bias towards deletion in bacteria[65,66]) and lost from bacterial populations by genetic drift (or, for costly genes, by negative selection). To test whether the genomes of co-diversified symbionts have experienced laboratory-specific expansions or contractions of specific gene families, we performed phylogenetic ANOVA for each gene family in each co-diversifying symbiont clade containing MAGs from both wild and laboratory mice (Supplementary Information). These analyses asked whether the genomes of multiple, phylogenetically independent symbiont lineages have gained or lost—convergently or in parallel—the same gene families in response to the transition from the wild into captivity. These analyses provided a rank-order list of genes enriched or depleted in MAGs from laboratory house mice relative to wild house mice. No gene functions were significantly enriched or depleted after false-discovery correction.

Lack of elevated genetic drift in non-co-diversifying GM clades

In addition to testing for elevated genetic drift in laboratory-mouse GM strains in co-diversifying clades ($r > 0.75$), we also tested for elevated genetic drift in laboratory-mouse GM strains in non-co-diversifying clades ($r < 0$) of similar phylogenetic depth to co-diversifying clades (*i.e.*, clades of congeneric MAGs in the distal 1/4th of bacterial phylogeny). These analyses did not support a significantly increased genetic drift, as measured by genome-wide elevation of dN/dS, in non-

codiversifying clades (paired t-test p-values = 0.0944 for tests based on genes under purifying selection, *i.e.*, genes for which dN/dS was < 1 in both wild-mouse and lab-mouse GM strains), contrasting the results observed for co-diversifying clades (Extended Data Table 8). Tests on individual non-co-diversifying clades also failed to strongly support increased genome-wide dN/dS in non-co-diversifying laboratory-mouse GM strains (paired t-test FDR-corrected p-values > 0.01 for all clades, and > 0.1 for all but 1 clade). A single clade (node 5493: an unclassified genus, CAG-1435, in the order Christensenellales) showed a marginally significant elevation in dN/dS in laboratory-mouse GM strains relative to WGM strains (paired t-test FDR-corrected p-value = 0.0241), and when all genes were tested, a marginally significant increase in dN/dS in the laboratory-mouse GM strains was observed (paired t-test p-values = 0.04065). This latter difference can be attributed to genes showing evidence of positive selection (dN/dS > 1) in laboratory-mouse GM strains but purifying selection (dN/dS < 1) in WGM strains, rather than increased genetic drift. Cumulatively, these results indicate that the significant elevation of genome-wide dN/dS (indicative of reduced *Ne* and increased genetic drift) in laboratory house mice observed for co-diversified GM strains was not apparent for non-co-diversified GM strains.

Elevated genetic drift in co-diversifying relative to non-co-diversifying clades in both laboratory and wild house mice.

Significant evidence of elevated genetic drift in laboratory GM strains was detected in co-diversifying clades but not non-co-diversifying clades (Tables S5, S8), suggesting that co-diversifying clades may be particularly predisposed to elevated genetic drift. Previous studies of insect endosymbionts have shown that bottlenecks during transmission of host-restricted symbionts can promote genetic drift[12,13], but the extent to which host-restriction of GM

symbionts in mammals promotes genetic drift has not been explored. To address this idea, we tested whether co-diversifying clades displayed stronger evidence of genetic drift than non-co-diversifying clades regardless of environment (laboratory or wild). We compared the distributions of per-gene log(dN/dS) values between co-diversified and non-co-diversified clades in both the laboratory and the wild. Results indicated significant elevation of genetic drift, as indicated by elevated genome-wide dN/dS, in co-diversified relative to non-co-diversified GM clades (Extended Data Figure 7) in both the laboratory (Extended Data Figure 7A) and the wild (Extended Data Figure 7B). These findings suggest that the host restriction of co-diversified clades predisposes these lineages to stronger genetic drift, which can be further enhanced by transitions from the wild to the laboratory environment (Fig. 3).

Removal of co-diversifying ASVs reduces signal of competitive advantage for wildling microbiota

To test whether ASVs of wildling or laboratory origin within co-diversifying taxa displayed a disproportionately strong competitive differential (compared to all ASVs), as suggested by Extended Data Figure 8, we compared results of beta-diversity analyses based on the complete dataset with those based on the complete dataset minus the ASVs used in analyses whose results are shown in Fig. 4. This comparison allowed us to test whether the removal of these ASVs belonging to co-diversifying taxa reduced the measured competitive differential between wildling and laboratory microbiota, as expected if co-diversifying laboratory GM strains have acquired increased genetic load. Indeed, removing these ASVs led to weaker signal of competitive advantage for wildling microbiota. On average, when all ASVs were included, the Dice beta-diversity differential between GF day 7–14 versus laboratory microbiota and GF 7–14

versus wildling microbiota was 0.237 (favoring wildling microbiota), whereas when only the subsetted ASVs (as defined in Fig. 3 and Extended Data Figure 8) were included this differential reduced to 0.22 (the difference in differential of only ~0.01, given that the subsetted ASVs constituted 105 of 2640 total ASVs. These findings indicate that laboratory-derived GM strains belonging to co-diversifying taxa displayed disproportionately stronger evidence for reduced fitness in these experiments than did other laboratory-derived GM strains, mirroring results presented in Extended Data Figure 8. These findings further support increased genetic load in laboratory-derived GM strains in co-diversifying taxa.

Supplemental References

59. Moran, N. A., Munson, M. A., Baumann, P. & Ishikawa, H. A molecular clock in endosymbiotic bacteria is calibrated using the insect hosts. *Proc. R. Soc. Lond. Ser. B: Biol. Sci.* **253**, 167–171 (1993).

60. Ochman, H., Elwyn, S. & Moran, N. A. Calibrating bacterial evolution. *Proc. Natl. Acad. Sci.* **96**, 12638–12643 (1999).

61. Ochman, H. & Wilson, A. C. Evolution in bacteria: Evidence for a universal substitution rate in cellular genomes. *J. Mol. Evol.* **26**, 74–86 (1987).

62. Duchêne, S. *et al.* Genome-scale rates of evolutionary change in bacteria. *Microb. Genom.* **2**, e000094 (2016).

63. Menardo, F., Duchêne, S., Brites, D. & Gagneux, S. The molecular clock of *Mycobacterium tuberculosis. PLoS Pathog.* **15**, e1008067 (2019).

64. Rascovan, N. *et al.* Emergence and spread of basal lineages of *Yersinia pestis* during the Neolithic decline. *Cell* **176**, 295-305.e10 (2019).

65. Sela, I., Wolf, Y. I. & Koonin, E. V. Theory of prokaryotic genome evolution. *Proc. Natl. Acad. Sci.* **113**, 11399–11407 (2016).

66. Kuo, C.-H. & Ochman, H. Deletional bias across the three domains of life. *Genome Biol. Evol.* **1**, 145–152 (2009).
